# The Relation of Pain and Fear in Cardiac Disturbance

**Published:** 1905-06-17

**Authors:** 


### THE RELATION OF PAIN AND FEAR IN CARDIAC DISTURBANCE.

Dr. G. R. Wilson, in a paper read before the Medico-Chirurgical Society of Edinburgh, has described an interesting group of symptoms and suggested an analogy between them and the sensory symptoms observed in cases of cardiac disturbance. His attention was first directed to the subject on being consulted on the same day by two patients, one an elderly widow, the other a young girl, who both complained of the same symptom, namely, of awaking from sleep in the morning in a state of terror. The fear was paroxysmal, transitory, and was accompanied by trembling, perspiration, and often by a sinking sensation. In the case of the elder patient there was mitral regurgitation of old standing, but this was the only case observed by the writer where paroxysms of this nature were associated with an organic cardiac lesion. Such terrors are more commonly met with in the subjects of nervous affections of the heart, especially the toxic conditions produced by alcohol and tobacco. The essential condition seems to be some alteration of the cardiac rhythm in the direction of acceleration or slowing or a combination of these; but there is reason to think that inhibitory disturbances prove most efficient in producing the attacks of fear. The nature of the fear varies, and is rarely related directly to the heart: for example, although the patient may fear that his heart is diseased or about to stop, he is more likely to dread having dispensed poison, having signed a cheque for too large an amount, or, in the case of an alcoholic patient, that he will be exposed and disgraced or will suffer from delirium tremens. These attacks present many analogies to the referred pains which occur in similar cases of cardiac disturbance, and are probably produced by a similar mechanism. Though it is impossible to demonstrate it, the paroxysms of terror must have an organic basis which is probably identical or closely allied to that concerned in the production of the referred pains of cardiac disturbance. The practical value of Dr. Wilson's views is the comfort which is afforded to the patient by an attempt at rational explanation.

